# Surgical procedures suppress autophagic flux in the kidney

**DOI:** 10.1038/s41419-021-03518-w

**Published:** 2021-03-05

**Authors:** Carolyn N. Brown, Daniel Atwood, Deepak Pokhrel, Sara J. Holditch, Christopher Altmann, Nataliya I. Skrypnyk, Jennifer Bourne, Jelena Klawitter, Judith Blaine, Sarah Faubel, Andrew Thorburn, Charles L. Edelstein

**Affiliations:** 1 Division of Renal Diseases and Hypertension, University of Colorado at Denver, Aurora, CO USA; 2 Electron Microscopy Center, University of Colorado at Denver, Aurora, CO USA; 3 Department of Anesthesiology, University of Colorado at Denver, Aurora, CO USA; 4 Department of Pharmacology, University of Colorado at Denver, Aurora, CO USA

**Keywords:** Autophagy, Kidney

## Abstract

Many surgical models are used to study kidney and other diseases in mice, yet the effects of the surgical procedure itself on the kidney and other tissues have not been elucidated. In the present study, we found that both sham surgery and unilateral nephrectomy (UNX), which is used as a model of renal compensatory hypertrophy, in mice resulted in increased mammalian target of rapamycin complex 1/2 (mTORC1/2) in the remaining kidney. mTORC1 is known to regulate lysosomal biogenesis and autophagy. Genes associated with lysosomal biogenesis and function were decreased in sham surgery and UNX kidneys. In both sham surgery and UNX, there was suppressed autophagic flux in the kidney as indicated by the lack of an increase in LC3-II or autophagosomes seen on immunoblot, IF and EM after bafilomycin A1 administration and a concomitant increase in p62, a marker of autophagic cargo. There was a massive increase in pro-inflammatory cytokines, which are known to activate ERK1/2, in the serum after sham surgery and UNX. There was a large increase in ERK1/2 in sham surgery and UNX kidneys, which was blocked by the MEK1/2 inhibitor, trametinib. Trametinib also resulted in a significant decrease in p62. In summary, there was an intense systemic inflammatory response, an ERK-mediated increase in p62 and suppressed autophagic flux in the kidney after sham surgery and UNX. It is important that researchers are aware that changes in systemic pro-inflammatory cytokines, ERK1/2 and autophagy can be caused by sham surgery as well as the kidney injury/disease itself.

## Introduction

Compensatory renal hypertrophy is an important consequence in both glomeruli and tubules following partial or complete UNX performed for renal cancer or for living kidney donors. Excessive compensatory renal hypertrophy can be a maladaptive response that leads to further nephron damage, tubular atrophy, interstitial fibrosis, loss of kidney function and chronic kidney disease^1^. It is well known that pS6, a marker of mTORC1, increases as early as 30 min after UNX and that mTORC1 inhibition with rapamycin can blunt UNX-induced renal hypertrophy^2^. mTOR-mediated phosphorylation of the transcription factor EB (TFEB), a master regulator of lysosomal biogenesis^3^, occurs at the lysosomal surface and controls the subcellular localization and activity of TFEB. Thus, the effect of UNX on lysosomal biogenesis and function, which are tightly tied to mTOR function, was determined.

As mTOR and lysosomal function are crucial to autophagy, autophagic flux was determined in vivo in the kidney. LC3-II, a marker of autophagosomes, was measured with and without the lysosomal inhibitor bafilomycin A1 (BafA1). According to the 2016 guidelines for the use and interpretation of assays for monitoring autophagy, if the basal increase in LC3-II is due to increased autophagosome production, then it is expected that lysosomal inhibition will further increase LC3-II (i.e., increased autophagic flux)^4^. Alternatively, if the increase in LC3-II is due to a block in autophagosome-lysosome fusion or a defect in autophagosome degradation by the lysosome, then lysosomal inhibition would not affect LC3-II expression. We measured the increase in LC3-II after lysosomal inhibition and used it to determine the effect of UNX on autophagic flux. The amount of p62/SQSTM1, a marker of autophagic cargo, is another method of determining autophagic flux as p62 can be destroyed by the lysosome much like LC3. In general, an increase in p62 indicates suppressed autophagic flux. p62 was measured in the kidney as an additional marker of autophagic flux^5^. Here, we demonstrated that there was suppressed flux which was associated with lysosomal abnormalities following both sham surgery and unilateral nephrectomy.

## Results

### Unilateral nephrectomy and sham surgery-dependent mTOR activation are attenuated by rapamycin

**Fig. 1:**
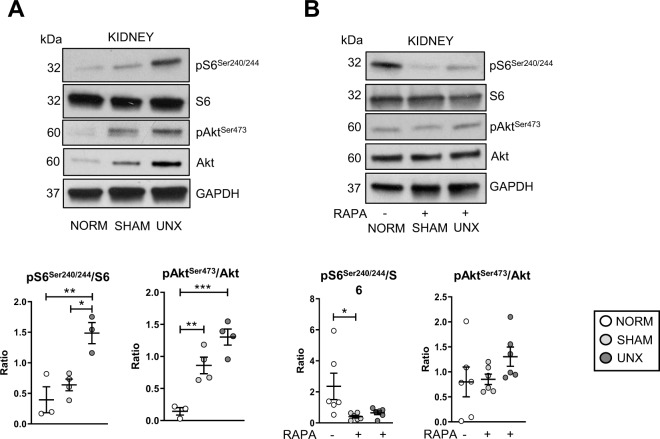
Increased mTORC1 (pS6Ser240/244) after UNX and mTORC2 (pAktSer473) after sham surgery and UNX in the kidney is attenuated by rapamycin. Mice underwent either no surgical manipulations (NORM), sham surgery (SHAM), or unilateral nephrectomy (UNX) and after 2 h the contralateral kidney was harvested. **A** Representative immunoblots and relative densitometry is shown for mTORC1 (pS6^Ser240/244^) and mTORC2 (pAkt^Ser473^) substrates in the kidney. *P < 0.05, **P < 0.01, ***P < 0.001. RDU = relative densitometry units corrected for GAPDH. **B** Representative immunoblots and relative densitometry is shown for mTORC1 (pS6) and mTORC2 (pAkt^Ser473^) substrates in the kidney after treatment of mice with rapamycin (RAPA). *P < 0.05, **P < 0.01. RDU = relative densitometry units.

The ratio of pS6^Ser240/244^ to total abundance of S6 protein was significantly increased in the kidney after UNX vs. normal and sham surgery (Fig. 1a), consistent with published studies^6,7^. pAkt^Ser473^, a marker of mTORC2, was significantly increased after both sham surgery and UNX (Fig. 1a). These data indicated that there was increased mTORC2 activation following both sham surgery and UNX, but increased mTORC1 only after UNX.

Rapamycin resulted in a significant decrease in pS6^Ser240/244^ in the kidney after sham surgery and UNX and blunted the increase in pAkt^Ser473^ (Fig. 1b). While rapamycin is known to be an indirect mTORC1 inhibitor, our results confirm previous studies that rapamycin can also inhibit mTORC2^8^.

### Increased mTOR after sham surgery and unilateral nephrectomy is associated with a lysosomal defect

**Fig. 2:**
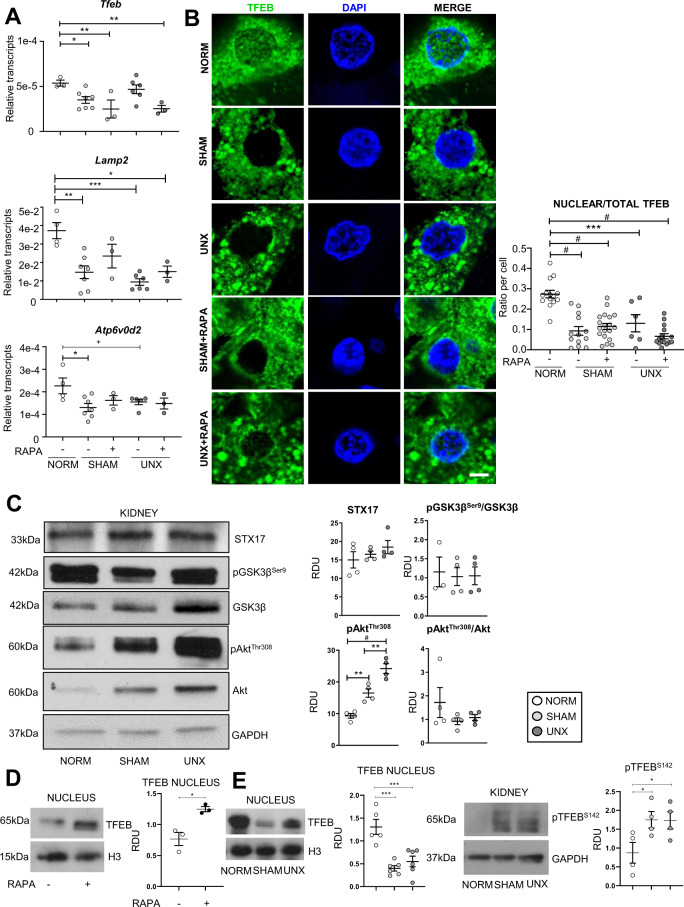
Sham surgery and unilateral nephrectomy are associated with a lysosomal defect. Mice underwent either no surgical manipulations (NORM), sham surgery (SHAM), or unilateral nephrectomy (UNX) and after 2 h the contralateral kidney was harvested. **A** qPCR analysis of TFEB-downstream genes *Tfeb*, *Atp6v0d2*, and *Lamp2*. **B** Representative proximal tubular cell images of TFEB nuclear versus cytoplasmic localization with quantification showing readings in duplicate for each mouse (Scale bar = 5 µm). **C** Immunoblot analysis for STX17, pGSK3β^Ser9^, GSK3β, pAkt^Ser308^ and Akt. **D** Immunoblot analysis for TFEB in nuclear extracts from rapamycin -treated (RAPA) mice. **E** Immunoblot analysis for TFEB in nuclear extracts from NORM, SHAM and UNX mice. **F** Immunoblot analysis for pTFEB^S142^ in whole kidney extracts from NORM, SHAM and UNX mice. Quantification is corrected for GAPDH unless otherwise indicated. ^+^*P* = 0.07, *P < 0.05, **P < 0.01, ***P < 0.001, ^#^*P* < 0.0001. RDU = relative densitometry units corrected for GAPDH. H3 = Histone H3.

mTOR is known to regulate TFEB, a master regulator of lysosomal biogenesis^3,9^ Quantitative polymerase chain reaction (qPCR) analysis for *Tfeb* and known TFEB-downstream genes *Atp6v0d2*, a vATPase subunit localized to the lysosomal membrane involved in lysosomal acidification^10^ and *Lamp2*^11^, a lysosomal-associated membrane protein, was performed. There were decreased *Tfeb*, *Atp6v0d2*, and *Lamp2* transcripts in sham surgery and UNX vs. normal kidneys (Fig. 2a). On immunofluorescence of tubular cells in the kidney cortex, there was less nuclear localization of TFEB after sham surgery and UNX vs. normal kidneys (Fig. 2b). Transcription of *Tfeb*-downstream genes and TFEB nuclear localization were not rescued by rapamycin (Fig. 2a, b), indicating that there may be additional mTOR-independent regulation of TFEB. p-Akt^Thr308^, that represses TFEB nuclear translocation independently of mTOR^12^, was increased in sham surgery and UNX (Fig. 2c). However, total Akt was also increased and when p-Akt^Thr308^ was corrected for total Akt, the increase was not significant (Fig. 2c). Syntaxin 17 (STX17), a vesicle docking protein essential for complete autophagosome-lysosome fusion^13^, and phosphorylated glycogen synthase kinase 3 beta (p-GSK3β), that activates a nuclear export signal for TFEB^14^, were not different between groups (Fig. 2c). TFEB regulates the expression of genes that encode autophagy-related proteins (Atg)^4^. The autophagy-related proteins, Atg3 and Atg7 play an important role in the LC3 lipidation process that is essential for autophagosome formation^4^ Atg3 was increased in sham surgery and UNX and Atg7 did not change in sham surgery and UNX (Supplementary Fig. S1) suggesting that the decrease in TFEB in the nucleus in sham surgery and UNX did not decrease the expression of Atg7 and 3.

TFEB was measured by immunoblot analysis in nuclear and cytoplasmic fractions of whole kidney from vehicle and rapamycin-treated mice and normal, sham surgery and UNX mice. mTOR inhibition is known to result in a translocation of TFEB to the nucleus^15^. As a positive control, we determined that there was an increase in TFEB in the nucleus in mice treated with rapamycin 10 mg/kg/day via I.P. injection for five days compared to vehicle (Fig. 2d). In agreement with the IF data, there was a decrease in TFEB in the nuclear fraction in sham surgery and UNX compared to normal (Fig. 2e). On immunoblot analysis, there was no difference in TFEB in the cytoplasmic fractions between the normal, sham and UNX groups (data not shown). TFEB is phosphorylated by mTOR on S142 and S211 serine residues, which play a crucial role in determining TFEB subcellular localization^16^. When these serines are phosphorylated, TFEB is mainly cytosolic and inactive^16^. In whole kidney extracts, there was an increase in pTFEB^S142^ in sham surgery and UNX compared to normal controls (Fig. 2f) which is compatible with the cytosolic localization of TFEB with sham surgery and UNX.

### Autophagic flux in the normal kidney in vivo

**Fig. 3:**
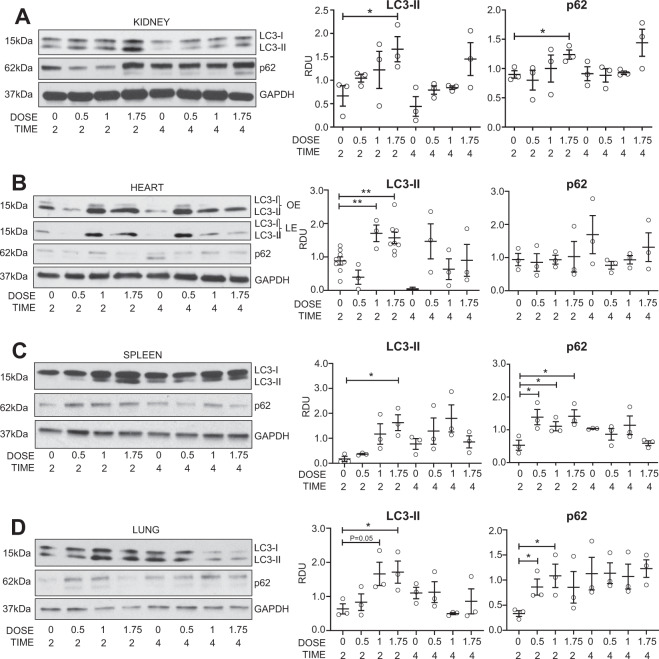
Autophagic flux in the normal kidney in vivo. A dose (mg/kg) and time (hours) study was performed for the lysosomal inhibitor, BafA1, in vivo. LC3-II and p62 were measured by immunoblot analysis in the **A** kidney, **B** heart, **C** spleen and **D** lungs. *P < 0.05, **P < 0.01. OE = over exposure, LE = light exposure, RDU = relative densitometry units corrected for GAPDH.

mTOR and lysosomal function that are crucial to autophagy, were affected by sham surgery and UNX. Thus, we hypothesized that sham surgery and UNX would suppress autophagic flux. To determine the optimal BafA1 dose and timing to use in vivo, mice were injected with increasing doses of BafA1. There was a significant increase in LC3-II and increase in p62 in the normal kidney after 2 h of treatment with 1.75 mg/kg BafA1 (Fig. 3a). Administration of 1.75mg/kg BafA1 also resulted in significantly increased LC3-II in the heart, spleen and lung (Fig. 3b–d). LC3-II was corrected for GAPDH. It has been recommended that the amount of LC3-II be compared to actin or GAPDH and not to LC3-I and that conclusions about autophagosome maturation not be made by comparing the amount of LC3-II to LC3-I^4^. The reason is that LC3-I is technically less sensitive to detection by antibodies that also detect LC3-II, LC3-I is labile and more sensitive to freeze-thawing and degradation in SDS buffer and the amount of LC3-I is cell and stress specific. Next, autophagic flux was measured in the kidney and heart after sham surgery and UNX.

### Autophagic flux is suppressed in the kidney after sham surgery and UNX

**Fig. 4:**
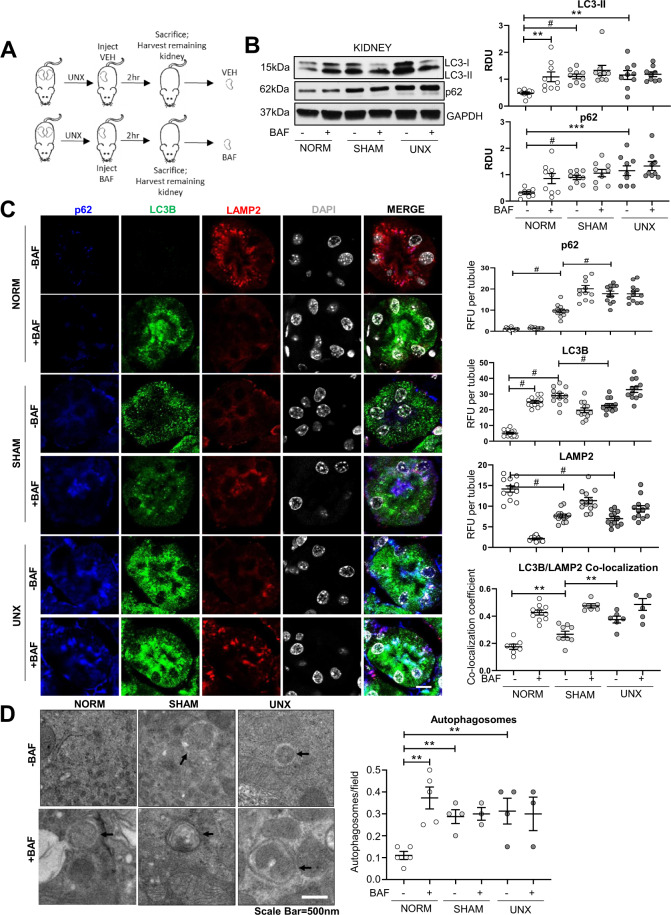
Suppressed autophagic flux after sham surgery and UNX in the kidney. **A** Mice underwent either no surgical manipulations (NORM), sham surgery (SHAM), or unilateral nephrectomy (UNX). Each mouse was treated with either vehicle (VEH) or BafA1 (BAF) and after 2 h the contralateral kidney was harvested. **B** Immunoblot analysis of LC3-II and p62 in the kidney with representative densitometry is demonstrated (*n* = 9 per group). Immunoblots were corrected for the endogenous control, GAPDH. RDU = relative densitometry units corrected for GAPDH. **C** Immunofluorescence analysis of punctate LC3B, LAMP2, and p62 per tubule in the kidney with quantification showing readings in duplicate for each mouse. RFU = relative fluorescence units. **D** Transmission electron microscopy for autophagosomes with quantification is demonstrated (arrows=autophagosome). Autophagosomes are quantified per 10 µm^2^ field. Scale Bar = 500 nm. **P < 0.01, ***P < 0.001, ^#^*P* < 0.0001.

Autophagic flux was measured in the kidney using the method described above (2 h of treatment with 1.75 mg/kg BafA1). Mice underwent either no surgery, sham surgery, or UNX, received either BafA1 or vehicle, and were sacrificed after 2 h (Fig. 4a). LC3-II did not increase with BafA1 after either sham surgery or UNX and p62 was increased in the kidney after sham surgery and UNX vs. normal mice (Fig. 4b). To test if sham surgery affects autophagic flux using independent methods and to determine the localization of autophagoooosomes in the kidney, IF and EM analysis for autophagosomes were performed and indicated changes in renal tubules. p62 was significantly increased after both sham surgery and UNX vs. normals on IF analysis (Fig. 4c). As the addition of phosphatidylethanolamine to LC3-I (i.e., LC3-II) cannot be differentiated by molecular weight on IF analysis, punctate LC3B was used as a measure of autophagosomes. LC3B puncta increased after BafA1 in normal kidneys (Fig. 4c). Significantly increased basal LC3B puncta were detected in both sham surgery and UNX vs. normal and were not further increased by BafA1 on IF analysis (Fig. 4c). There was a significant decrease in LAMP2 after both sham surgery and UNX (Fig. 4c). On EM analysis there was a decrease in the number of lysosomes after both sham surgery and UNX (Supplementary Fig. S2). Co-localization of LAMP2 and LC3B was increased after sham surgery and further increased after UNX similar to what was observed after BafA1 administration (Fig. 4c). These data indicate an increased number of autolysosomes that were unable to break down the autophagosomal cargo and membrane (i.e., LC3-II and p62) suggesting that autophagosome formation was not compromised and that surgery blocks autophagy by causing a defect in the later stages of autophagy^17,18^. EM analysis revealed significantly increased autophagosomes after BafA1 in normal mice (Fig. 4d). There was an increase in basal autophagosomes in both sham surgery and UNX compared to normal, but BafA1 did not induce further increase autophagosomes. These results, which were obtained from 3 independent techniques, indicate that autophagy in renal tubules was suppressed after sham surgery and UNX in the kidney. Further, rapamycin, which did not rescue lysosomal defects (Fig. 2), did not restore autophagic flux in the kidney after sham surgery or UNX (Supplementary Fig. S3).

### Metabolomics analysis

Lysosomal defects are known to have effects on availability of amino acids and other metabolites^19^. Thus, metabolomics analysis was performed to determine whether the lysosomal defect and suppressed flux were associated with an altered renal metabolome. Metabolomics analysis was performed on the kidney taken 2 h after sham surgery or UNX (Supplementary Fig. S4A–C). Interestingly, although lysosomal deficiency was similar in both sham surgery and UNX, only fructose phosphate, glycine, and folate (Supplementary Fig. S5A) were affected similarly after sham surgery and UNX. There is little known about the effects of fructose phosphate, glycine, and folate on autophagy^20–22^ Most of the metabolites measured were differentially effected by either sham surgery or UNX (Supplementary Fig. S5B–F) or were unchanged by sham surgery or UNX (Supplementary Fig. S6).

### Autophagic flux is suppressed in the heart after UNX

The surgical procedure has been shown to affect monocyte subset kinetics in a murine model of myocardial infarction^23^ Thus, we determined whether the effects seen following sham surgery and UNX were unique to the kidney or involved the heart as well. BafA1 resulted in a significant increase in LC3-II in normal hearts (Fig. 3b). There were no significant changes in activation of mTORC1 (pS6 Ser240/244) or mTORC2 activation (pAktSer473) in the heart after sham surgery or UNX (Supplementary Fig. S7A). Unexpectedly, flux (the increase in LC3-II with BafA1) in heart was completely suppressed after UNX, but not sham surgery (Supplementary Fig. S7C). Although rapamycin inhibited mTORC1/2 in the heart (Supplementary Fig. S7B), it did not rescue autophagic flux after UNX (Supplementary Fig. S7D).

### Both the sham surgery and bilateral renal ischemia/reperfusion suppressed autophagic flux in the heart

**Fig. 5:**
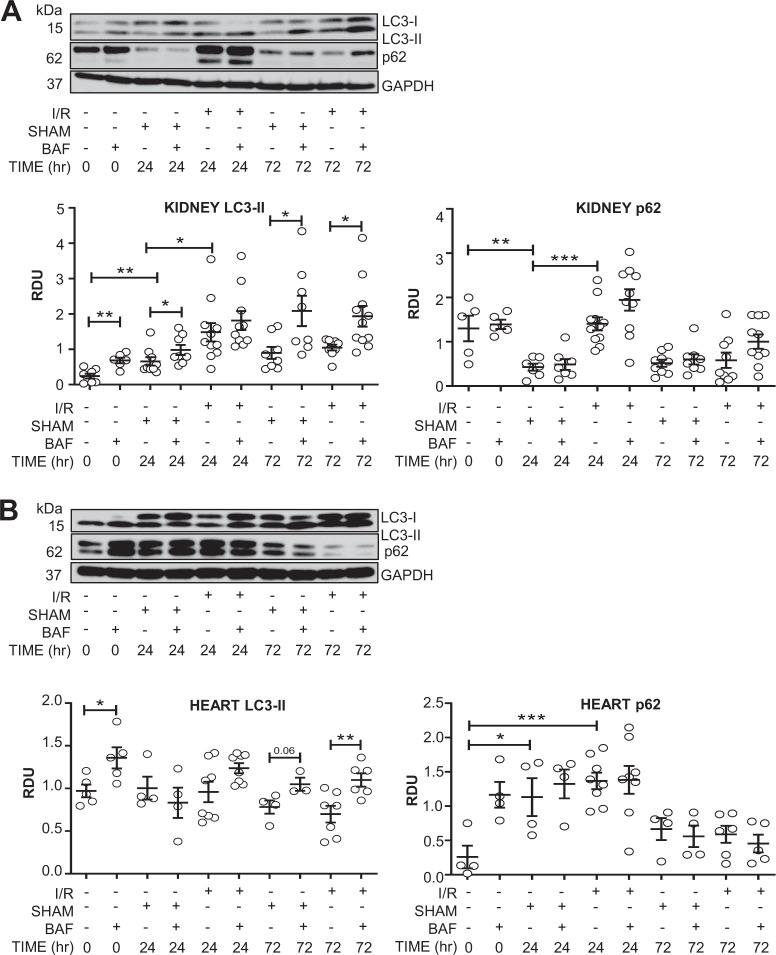
Both sham surgery and bilateral renal ischemia/reperfusion (24â€‰h) suppressed autophagic flux in the kidney and heart. Autophagic flux normalizes at 72 h. Mice underwent either no surgical manipulations, sham surgery (SHAM), or bilateral renal ischemia/reperfusion (I/R). After 24â€‰h or 72â€‰h, each mouse was treated with either vehicle or BafA1 (BAF) and after 26â€‰h or 74â€‰h of reperfusion, the contralateral kidneys and heart were harvested. Immunoblot analysis of LC3-II and p62 in the kidney (**A**) and the heart (**B**) with representative densitometry is demonstrated. RDUâ€‰=â€‰relative densitometry units corrected for GAPDH. *Pâ€‰<â€‰0.05, **Pâ€‰<â€‰0.01, ***Pâ€‰<â€‰0.001.

To determine whether other surgical procedures affect autophagic flux, a bilateral renal ischemia/reperfusion (I/R) model of AKI was performed. Mice underwent either no surgery, sham surgery, or I/R for 24 or 72 h. At 24 h after I/R there was a lack of increase in LC3-II with BafA1 suggestive of decreased autophagic flux (Fig. 5a). However, sham I/R surgery in the kidney resulted in an increase in LC3-II with bafilomycin and a decrease in p62 at 24 h suggesting that sham I/R surgery does not suppress autophagic flux (Fig. 5a). Interestingly, in the heart, both sham I/R surgery and I/R at 24 h resulted in suppressed autophagic flux as evidenced by the lack of increase in LC3-II with bafilomycin and an increase in p62 (Fig. 5b). At 72 h after both sham surgery and I/R, autophagic flux normalized in kidney and heart (Fig. 5a, b). These data suggest that sham surgery for renal I/R had different effects on autophagic flux in kidney compared to heart and consistently normalized by 72 h after the procedure.

### Pro-inflammatory cytokine “storm” in the serum after sham surgery and UNX

**Fig. 6:**
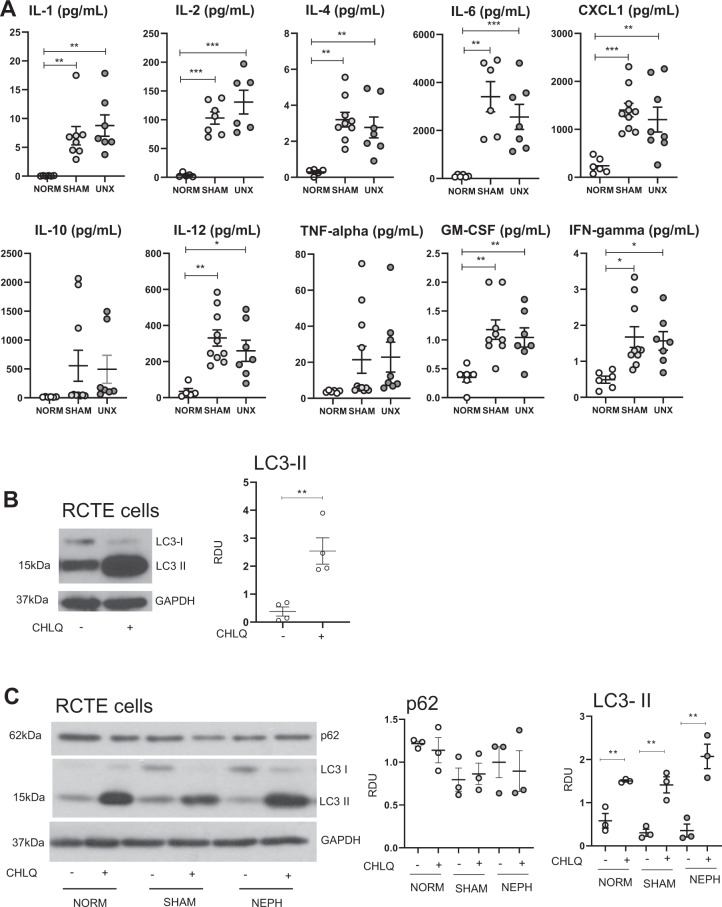
Pro-inflammatory cytokine “storm” in the serum after sham surgery and UNX. Serum from normal, sham surgery or UNX mice did not suppress autophagic flux in RCTE cells. Mice underwent either no surgery, sham surgery, or UNX and were sacrificed after 2 h. **A** A panel of pro-inflammatory cytokines was measured in the serum: IL-1β, IL-2, IL-4, IL-6, CXCL1 (also known as IL-8 in humans and KC in mice), IL-10, IL-12, GM-CSF, IFN-γ, TNF-α. RCTE cells were treated with **B** normal medium or **C** medium containing 10% serum from normal mice, sham surgery or UNX mice. Immunoblot analysis of LC3-II and p62 in RCTE cells with or without chloroquine (CHLQ) with representative densitometry is demonstrated. Immunoblots were corrected for the endogenous control, GAPDH. *P < 0.05, **P < 0.01, ***P < 0.001.

As sham surgery and UNX resulted in changes in autophagy in the kidney and heart, we reasoned that a factor in the serum might be causing these effects. Sham surgical procedures have been shown to cause an inflammatory response^24,23^ and production/release of pro-inflammatory cytokines, which are known to affect autophagy^25^. Thus, a panel of pro-inflammatory cytokines was measured in the serum. Mice underwent either no surgery, sham surgery, or UNX and were sacrificed after 2 h. There were massive increases in the serum in IL-1β (up to 50-fold), IL-2, IL-4, IL-6 (up to 100-fold), CXCL1 (also known as IL-8 in humans and KC in mice), IL-12, GM-CSF, IFN-γ in sham surgery and UNX compared to no surgery (Fig. 6a).

To determine whether the pro-inflammatory cytokine “storm” measured in the serum in sham surgery and UNX had an effect on autophagic flux, renal cortical tubular epithelium (RCTE) cells were treated with normal medium or medium containing 10% serum from normal mice, sham surgery or UNX mice. RCTE cells treated with normal medium had a large increase in LC3-II with chloroquine indicating autophagic flux (Fig. 6b). RCTE cells treated with medium containing 10% serum from normal, sham surgery or UNX mice also had a large increase in LC3-II with chloroquin (Fig. 6c). RCTE cells treated with medium containing 10% serum from normal, sham surgery or UNX mice had no change in p62 (Fig. 6c). These data indicate that pro-inflammatory cytokine-rich serum from mice that had sham surgery or UNX did not suppress autophagic flux in RCTE cells.

### ERK1/2 inhibition attenuated the increase in p62 seen after sham surgery and unilateral nephrectomy

**Fig. 7:**
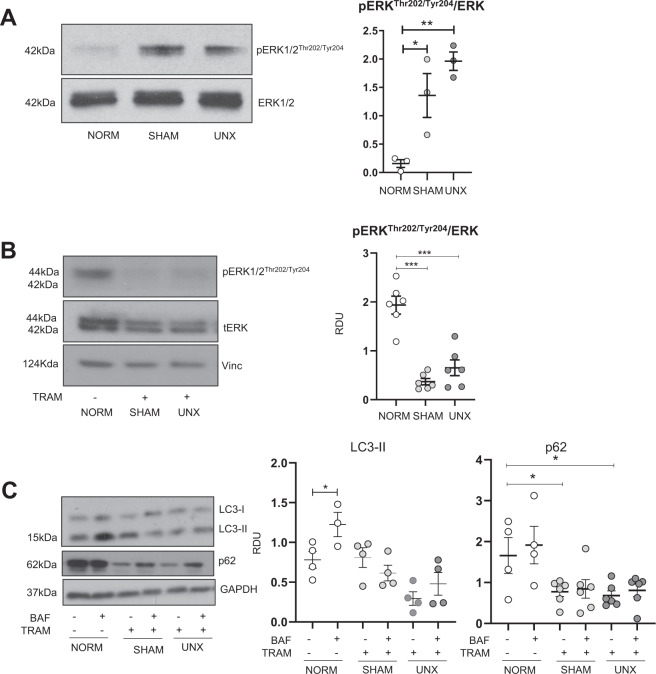
ERK1/2 inhibition attenuates increased p62 seen after sham surgery and UNX. Mice were treated with either Trametinib (1 mg/kg/d IP) or vehicle (3% DMSO in saline) for 3 days. Mice then underwent either no surgical manipulations (NORM), sham surgery (SHAM), or unilateral nephrectomy (UNX). Each mouse was treated with either vehicle (VEH) or BafA1 (BAF) and after 2 h the contralateral kidney was harvested. **A** pERK1/2 was significantly increased in sham surgery and UNX vs. normal kidneys. **B** Trametinib resulted in a near disappearance of pERK1/2 in sham surgery and UNX kidneys. **C** Trametinib resulted in a significant decrease in p62 in sham surgery and UNX kidneys compared to normal kidneys, but did not change the suppressed autophagic flux that was seen in sham surgery and UNX kidneys. Immunoblot analysis of LC3-II and p62 in the kidney with representative densitometry is demonstrated. RDU= relative densitometry units corrected for GAPDH. *P < 0.05, **P < 0.01, ***P < 0.001.

Pro-inflammatory cytokines have been shown to upregulate pERK1/2^26,27^ which can in turn regulate the nuclear localization and activity of TFEB^9^ and autophagy^28^. pERK1/2 was significantly increased in sham surgery and UNX vs. normal kidneys (Fig. 7a). Mice were treated with the MEK1/2 inhibitor, Trametinib (1 mg/kg/d IP) that is also a potent ERK1/2 inhibitor, for 3 days and then sham surgery or UNX was performed. Trametinib resulted in a near disappearance of pERK1/2 in sham surgery and UNX kidneys (Fig. 7b). Trametinib resulted in a significant decrease in p62 in sham surgery and UNX kidneys compared to normal kidneys, but did not change the suppressed autophagic flux that was seen in sham surgery and UNX kidneys (Fig. 7c).

## Discussion

The major findings of this study are: (1) Increased mTORC1/2 signaling associated with TFEB and lysosomal abnormalities and suppressed autophagic flux in the kidney after both sham surgery and UNX, (2) Large increases in pro-inflammatory cytokines in the serum, and pERK1/2 and p62 in the kidney after sham surgery and UNX. The MEK1/2 inhibitor, trametinib, that also potently inhibits ERK, resulted in decreased p62 in the kidney after sham surgery and UNX. (3) Both sham surgery and renal I/R suppressed autophagic flux in the kidney and heart.

Based on the known role of mTORC1 in compensatory renal hypertrophy^2,7^ our data showing increased mTORC1/2 after sham surgery and UNX, and the known role of mTOR in lysosomal biogenesis^3^, the effect of UNX on TFEB and lysosomal function was determined. In general, lysosomal defects are characterized by decreased lysosomal biogenesis or impaired lysosomal function (i.e., pH, activity of lysosomal enzymes). LAMP2, a marker of lysosomes, was decreased in both sham surgery and UNX, suggesting decreased lysosomal biogenesis. On qPCR analysis *Lamp2* mRNA was also found to be decreased, suggesting regulation at the transcriptional level. As TFEB is a master regulator of lysosomal biogenesis^9^, changes in TFEB were studied. *Tfeb* mRNA in the kidney was decreased after sham and UNX. There was less nuclear localization of TFEB protein, where it induces transcription of target genes, in the kidney after sham surgery and UNX. On nutrient depletion and under abnormal lysosomal storage conditions TFEB is known to translocate to the nucleus, resulting in the transcription of its target genes such as vesicular ATPases (vATPases) that contribute to lysosomal acidification and function^9^. In this regard, we found mRNA expression of *Atp6v0d2*, a vATPase subunit, to be decreased after sham surgery and UNX. These data suggest that there is a lysosomal defect (decreased lysosomal biogenesis, decreased lysosomal ATPase) in sham surgery and UNX.

An increase in LC3B/LAMP2 co-localization often indicates an activated autophagy-lysosome pathway. Co-localization of LC3B and LAMP2 was increased after sham surgery and UNX, indicating that there was likely no defect in trafficking. In the context of autophagy, defects in lysosome-autophagosome trafficking and fusion can interfere with autophagic flux. Expression of STX17, which is involved in autophagosome-lysosome fusion, was unchanged, indicating that there was likely no defect in fusion^13^. These data indicate an increased number of autolysosomes that were unable to break down the autophagosomal cargo and membrane (i.e., LC3-II and p62) suggesting that autophagosome formation or autophagosome-lysosome fusion was not compromised and that surgery blocks autophagy by causing a defect in the later stages of autophagy^17,18^.

Next, upstream mechanisms of the lysosomal defect seen after sham surgery and UNX were studied. Transcription of TFEB-downstream genes and TFEB nuclear localization were not rescued by rapamycin, suggesting an mTORC1/2-independent pathway. pERK1/2, that is known to decrease nuclear localization of TFEB and activity of TFEB^9^, was increased in sham surgery and UNX. Akt, has been shown to affect lysosomal biogenesis and function through inhibitory phosphorylation of TFEB^Ser467^^29^. In this regard, there was a large increase in pAkt^Ser473^ (mTORC2-dependent) but not pAkt^Thr308^/total Akt (mTORC2-independent) after sham surgery and UNX. However, rapamycin that inhibited the increase in mTORC1 (pS6^Ser240/244^) and mTORC2 (pAkt ^Ser473^), did not correct the lysosomal abnormalities. GSK3β, that activates a nuclear export signal for TFEB^14^, was not significantly affected by sham surgery or UNX. These data indicate that the suppressed flux seen after UNX may be due to mTORC1/2-independent lysosomal dysfunction associated with a large increase in pERK1/2. The idea that signaling in sham surgery and UNX affect TFEB function and induce lysosomal defects led us to determine the effect of sham surgery and UNX on autophagic flux in vivo.

Bafilomycin can initiate autophagosome formation via inhibition of mTORC1 by the Rag signaling molecule^30^. However, in sham surgery mTORC1 (as determined by pS6 immunoblot analysis) was not decreased and in UNX mTORC1 was increased. Excessive exposure time (more than 4 h) of BafA1 can also lead to non-specific effects^4^ such as proteasome inhibition^31^. Thus we used BafA1 (1.75 mg/kg) for 2 h that resulted in a significant, consistent, and reproducible increase in LC3-II in kidneys, heart, spleen, and lungs in BafA1-treated mice compared to vehicle-treated mice.

It is known that patients with AKI and patients undergoing surgical procedures especially abdominal surgery have large increases in serum cytokines and that this inflammatory response is associated with liver, lung and heart injury^32,33^. Mouse studies have been performed to better understand how surgical procedures cause a systemic inflammatory response and organ injury. There is a large increase in systemic pro-inflammatory cytokines in a mouse model of ischemic AKI that is associated with histological injury in the lung^24,34^ liver^35^ and heart^36^. However, the effect of sham surgery on systemic inflammation is less well known. The sham surgical procedure in a mouse model of myocardial infarction can cause a systemic monocyte-induced inflammatory response^23,37^ Another study also shows a systemic cytokine response by the surgical procedure to induce mouse myocardial infarction^38^. Our previous study in the same ischemic AKI model used in the present study, demonstrated that 9 cytokines increased as early as 2 h after sham operation compared to baseline^24^. IL-6, but not TNF-α, was increased by sham biliary obstruction surgery or sham partial hepatectomy surgery in mice^39,40^ In the present study, we show for the first time that there was a massive increase in pro-inflammatory cytokines in the serum in sham surgery and UNX. It is interesting to speculate whether the inflammatory response caused by sham surgery can cause distant organ injury. In a mouse ischemic AKI model, although there was a systemic inflammatory response caused by sham surgery, the sham surgery did not cause histological lung injury^24^. However, sham surgery for myocardial infarction resulted in a significant change in monocyte subset kinetics in the heart^23^. In future studies, it will be interesting to determine whether sham surgery alone can cause local and distant organ injury.

Pro-inflammatory cytokines have been shown to upregulate pERK1/2^26,27,27^ and ERK1/2 can result in an increase in p62^41^. There was a significant increase in p62 in sham surgery and UNX kidneys. p62 functions in both as an autophagy receptor and as a signaling molecule. p62 links cargo proteins with the autophagosome membrane and an increase in p62 is generally indicative of suppressed autophagic flux^35^. The increase in p62 was ERK1/2-dependent as trametinib inhibition of the large increase in pERK1/2 seen in sham surgery and UNX kidneys resulted in a decrease in p62.

There is increased ERK1/2 activation in surgery-induced AKI and inhibition of ERK results in protection against ischemic AKI^42,43^ Also in UNX, there is increased ERK1/2 in the remaining kidney^44^. The ERK1/2 pathway is known to regulate autophagy and the effect of ERK1/2 on autophagy is model and stimulus specific^45^. However, the effect of the ERK pathway on autophagy in the context of kidney injury or UNX is not well known. Compatible with our results that increased ERK1/2 in sham surgery and UNX is associated with decreased autophagic flux, it has been shown that increased ERK signaling decreases autophagic flux in pancreatic cancer cells^46^. Also, in ischemic AKI, melatonin resulted in decreased ERK1/2, increased autophagy and functional protection against AKI^47^.

The effect of sham surgery and UNX to suppress autophagic flux has not previously been described. This finding is important for researchers performing mouse studies for the following reasons: 1) Suppressed autophagy may account for mouse mortality that is often not reported, 2) Our results exposed a previously underappreciated impact of sham surgical procedures on lysosomal function, systemic cytokines, pERK1/2, p62 and autophagy. Of importance is that the increase in pro-inflammatory cytokines, pERK1/2 and suppressed autophagic flux caused by sham surgery may have implications for interpreting results of autophagy studies using animal models requiring sham surgery. Thus, caution should be taken with many animal studies where investigators are trying to study effects on autophagy on organ systems, even when the appropriate sham controls are included. Suppressed autophagy in the kidney and heart may also explain some of the morbidity seen in patients associated with surgery.

**Fig. 8:**
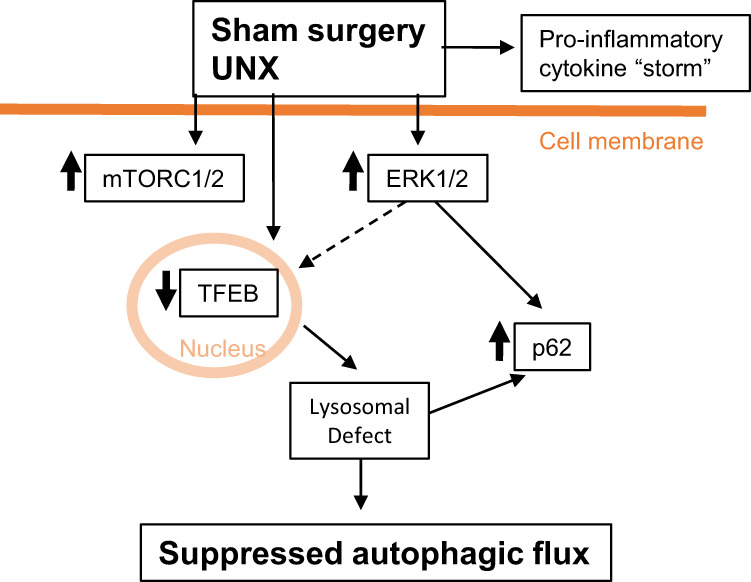
Surgical procedures suppress autophagic flux. Sham surgery and unilateral nephrectomy (UNX) resulted in an increase in pro-inflammatory cytokines in the serum. There was an increase in mTORC1/2 proteins and phosphorylated ERK1/2 (pERK1/2) in the kidney in sham surgery and UNX. The increase in pERK1/2 resulted in an increase in p62. ERK1/2 potentially inhibited (dotted line) TFEB nuclear localization and the lysosomal defect seen in sham surgery and UNX. A systemic cytokine “storm”, a large increase in pERK1/2, a lysosomal defect and suppressed autophagic flux caused by sham surgery may confound the interpretation of results of autophagy studies using animal models requiring surgical procedures.

In summary, a method to determine autophagic flux in vivo was developed and demonstrated that surgical procedures suppressed autophagic flux in kidney and heart. There was an increase in serum pro-inflammatory cytokines, and kidney pERK1/2 and p62 after sham surgery and UNX (Fig. 8). Trametinib, a MEK1/2 inhibitor that is also a potent inhibitor of ERK1/2, resulted in a decrease of p62 in sham surgery and UNX kidneys. Description of the effect of sham surgery to suppress autophagy in the kidney and heart is novel. A large increase in pERK1/2, a systemic cytokine “storm and suppressed autophagic flux caused by sham surgery may confound the interpretation of results of autophagy studies using animal models requiring surgical procedures (Fig. 8).

## Methods

### Drug dosing

5 mg of BafA1 (Cayman Chemical #11038) was dissolved in 1 mL DMSO and diluted to 1 mg/mL in PEG300. Rapamycin was prepared in 28% DMSO in PEG300 and injected IP at 0.5 mg/kg immediately before surgery.

### Animals

Wild type male C57BL/6J mice (#000664) at 10 weeks of age were purchased from Jackson Laboratories (Bar Harbor, ME, USA). Mice were randomized to receive either BafA1 or vehicle.

### UNX and sham surgery

A laparotomy was performed under isoflurane anesthesia. A silk suture was tied around renal artery/vein and ureter and the kidney was removed, followed by suturing of the muscle and skin. For sham surgery, the same procedure and anesthesia was used in a separate mouse; after maneuvering the kidney out of the body, the kidney was gently caressed with two cotton swabs for 2 min and returned to peritoneum. Normal control animals that did not have any surgical procedure received the same method of sacrifice as the UNX and sham surgery animals: isoflurane overdose followed by cervical dislocation. It was determined that 13 min of 1 liter per minute (LPM) oxygen and 2 LPM isoflurane anesthesia, which were used in the UNX and sham surgery animals, had no effect on autophagic flux (Supplementary Fig. S8A).

### Isolation of nuclear and cytosolic fractions

Nuclear and cytoplasmic fractions of whole kidney from vehicle and rapamycin-treated mice and normal, sham surgery and UNX mice were obtained using the NE-PER™ nuclear and cytoplasmic extraction kit (78833) by Thermo-Fisher (Waltham, MA, USA) following manufacturer’s instructions.

### Bilateral renal ischemia/reperfusion (I/R) surgery

Bilateral renal pedicle clamping for 29 min was performed as previously described^48^. Sham surgery consisted of the same procedure except that clamps were not applied. Normal control animals that did not receive any surgical procedure received the same method of sacrifice as the I/R and sham surgery animals: isoflurane overdose followed by cervical dislocation. It was determined that ketamine/xylazine, which was used in the bilateral ischemia reperfusion and sham surgery animals, had no effect on autophagic flux (Supplementary Fig. S8B).

### Immunoblot analysis

Immunoblots were performed as previously described^49^. Briefly, one piece of tissue was immersed in 750 μl tissue lysis buffer (1× RIPA, 1× protease inhibitor, 1× PMSF) and homogenized. Homogenate was centrifuged at 4 °C for 25 min at 15,000 r.p.m. and supernatant was taken for protein quantification by BioRad DC Protein Assay (Hercules, CA, USA). Samples were mixed with Laemmli Sample Buffer and boiled for 5 min. Samples were run on 4–15% gradient precast polyacrylamide gels. Proteins were then transferred to 0.45µm PVDF membranes, blocked with 2.5% evaporated milk, and probed with antibodies listed in Supplementary Table 1. Blots were developed by chemiluminescence and analyzed for densitometry using ImageJ. 20 µg of protein was loaded in each lane. Serial dilutions of the proteins were loaded to verify that 20 µg was in the linear range for quantitative Western blot detection.

### Immunofluorescence

Fixation of tissue, preparation of slides and immunofluorescence was performed as we have previously described^50^. Negative controls (secondary antibody only) were tested for each fluorophore used. Slides were imaged using an Olympus FV1000 confocal laser scanning microscope with a 100× oil objective. Images were taken of cortical tubular regions. Co-localization analysis was performed using Olympus FluoView software. Ratio of nuclear-localized protein was calculated by dividing the nuclear fluorescence intensity by the total fluorescence intensity of the same cell.

### qPCR analysis

RNA was isolated from kidney tissue using the Qiagen RNeasy kit as described by the manufacturer. cDNA was synthesized using Takara Bio RNA to cDNA kit as described by the manufacturer. 100ng of cDNA was used per reaction with BioRad SYBR green mix and primers as listed in Supplementary Table S2. Ultrapure DNase-/RNase-free water was used as a no template control (NTC). Expression of each gene was corrected for expression of β-actin.

### Transmission electron microscopy

Fixation of tissue, processing and sectioning and analysis was performed as described^51^. Sections were imaged on a FEI Tecnai G2 transmission electron microscope (Hillsboro, OR) with an AMT digital camera (Woburn, MA). At least 20 images of proximal renal tubules, where a brush border was clearly seen, were taken per sample. Images were quantified by multiple, independent, blinded observers. Exclusion/inclusion criteria for autophagosomes have been adapted from several sources and are described in Supplementary Table S3.

### Metabolomics

Kidney tissue samples were extracted according to a protocol by Yuan et al^52^ and analyzed using high-performance liquid chromatography-mass spectrometry (HPLC-MS). After homogenization in 80% methanol solution (volume/volume), samples were incubated for 6–8 h at −80 °C to allow for protein precipitation. Following repeated extraction, samples were centrifuged again and dried in a SpeedVac concentrator (Savant, ThermoFisher, Waltham, MA). Samples were reconstituted with 20 µl of water/methanol (80:20, volume/ volume) and were analyzed using high-performance liquid chromatography-mass spectrometry (HPLC-MS). Sample analysis was performed using an Agilent 1200 series HPLC system (Agilent Technologies, Palo Alto, CA) interfaced with an ABSciex 5500 hybrid triple quadrupole/linear ion trap mass spectrometer (Concord, ON, Canada) equipped with an electrospray ionization source operating in the positive/ negative switch mode. Once the data were acquired, MultiQuant (v2.1.1., ABSciex) software was used for data analysis of 216 unique metabolites. Metabolite peaks were normalized by tissue weight and total area integral of each sample prior to performance of statistical analyses. Analysis of metabolomics data was performed using MetaboAnalyst (metaboanalyst.ca). The Q1 (precursor ion) and Q3 (fragment ion) transitions, the metabolite names, dwell times and the appropriate collision energies for both positive and negative ion modes were adapted from Yuan et al. with several metabolite transitions added by our group. Q1 and Q3 transitions were set to unit resolution for optimal metabolite ion isolation and selectivity. In addition, the polarity switching (settling) time was set to 50 ms. In 1.42 s using a 3-ms dwell time, we were able to obtain 6–14 scans per metabolite peak. Eight μL of sample was injected onto an Amide XBridge HPLC column (3.5 μm; 4.6 mm inner diameter [i.d.] × 100 mm length) (Waters, Milford, MA). The mobile phases consisted of HPLC buffer A (pH = 9.0: 95% (vol/vol) water, 5% (vol/vol) acetonitrile, 20 mM ammonium hydroxide, 20 mM ammonium acetate) and HPLC buffer B: 100% acetonitrile. The HPLC settings were as follows: from 0 to 3 min, the mobile phase was kept at 85% B; from 3 to 22 min, the percentage of solvent B was decreased from 85% to 2% and was kept at 2% for additional 3 min. At minute 26, solvent B was increased again back to 85% and the column flushed for additional 7 min at 85% solvent B.

### Measurement of cytokines

A multiplex sandwich immunoassay was used to measure ten inflammatory cytokines (Meso Scale Discovery, MULTI-SPOT Assay System, V-plex Proinflammatory Panel-1 for mice, Catalog no: K15048D-1, Rockville, MD, USA).

### In vitro cell experiments

Human primary cells from the normal renal cortical tubular epithelium (RCTE), immortalized with ori-adeno-simian virus 40, were used, as previously described^53^. Briefly, cells were plated 18 h in advance. Once plates had reached 80% confluence, the medium was changed to one of the following: (1) normal medium, (2) medium containing 10% serum from normal mice, (3) medium containing 10% serum from sham surgery mice, (4) medium containing serum from UNX mice. Cells were then exposed to chloroquine 100 nm or vehicle for measurement of autophagic flux as described^54^. After 2 h, protein was isolated from cell lysates and immunoblotted for LC3-II and p62.

### Statistical analysis

The sample size was chosen based on our previous experience with similar mouse models and experimental design. All graphs and statistical analyses were managed using GraphPad Prism Software. The Students *t*-test was used to achieve statistical significance between two groups. Multiple group comparisons were performed using one-sided analysis of variance (ANOVA) with posttest according to Tukey. A *P* value of <0.05 was considered statistically significant. Values were expressed as scatter dot plots with each dot representing a separate animal with lines at means and SEM. In all analyses, SEMs were not significantly different between groups. The investigator was blinded to the group allocation when assessing the outcome.

## Supplementary information

Supplement
