# Supplementary material for: Surgical procedures suppress autophagic flux in the kidney
Source: Cell Death Dis. 2021 Mar 5;12(3):248. doi: 10.1038/s41419-021-03518-w (PMC7935862; doi:10.1038/s41419-021-03518-w)

**Supplementary Table S1. Antibodies used for immunoblot (IB) and immunofluorescence (IF) analyses.**

| **Manufacturer** | **Antibody** | **Catalog #** | **Dilution** |
| --- | --- | --- | --- |
| Cell Signaling Technology (Danvers, MA, USA) | LC3B | 2775 | 1:1000 IB  1:100 IF |
|  | p62/SQSTM1 | 5114 | 1:500 IB |
|  | pS6^Ser240/244^ | 5364 | 1:1000 IB |
|  | S6 | 2317 | 1:1000 IB |
|  | pAkt^Ser473^ | 9271 | 1:500 IB |
|  | Akt | 9272 | 1:1000 IB |
|  | pAkt^Thr308^ | 9275 | 1:1000 IB |
|  | pERK^Thr202/Tyr204^ | 9101 | 1:1000 IB |
|  | ERK | 9102 | 1:1000 IB |
|  | GAPDH | 2118 | 1:10,000 IB |
|  | Anti-Rabbit IgG HRP | 7074 | 1:5000 IB |
| Thermo Fisher Scientific (Waltham, MA, USA) | LAMP2 | PA1-655 | 1:100 IF |
|  | p62/SQSTM1 | MA5-27800 | 1:100 IF |
|  | STX17 | PA5-40127 | 1:1000 IB |
|  | Anti-rat  AlexaFluor546 | A11081 | 1:1000 IF |
|  | Anti-mouse IgG_2b_ AlexaFluor647 | A21242 | 1:1000 IF |
| Jackson ImmunoResearch (West Grove, PA, USA) | Anti-rabbit AlexaFluor488 | 111-545-144 | 1:500 IF |
| ProteinTech (Rosemont, IL, USA) | TFEB | 13372-1-AP | 1:1000 IB  1:50 IF |
| Sigma-Aldrich  (Burlington, MA, USA) | pTFEB^S142^ | ABE1971-I | 1:1000 IB |

**Supplementary Table S2. Primer sequences used for qPCR analysis.**

| **Gene** | **Forward primer** | **Reverse primer** |
| --- | --- | --- |
| *Actb* | 5’-GATGTATGAAGGCTTTGGTC-3’ | 5’-TGTGCACTTTTATTGGTCTC-3’ |
| *Tfeb* | 5’-ACTATGATGGGGAAGAACAG-3’ | 5’-GGTACTTGTACCTCCTTCTC-3’ |
| *Lamp2* | 5’-CTAATGGCTCAGCTTTCAAC-3’ | 5’-AAAGGTGTTGATCTGAAACG-3’ |
| *Atp6v0d2* | 5’-ATGGAGTTTACAAGCCTTTG-3’ | 5’-TTCATCTGTACCTCTCTCTC-3’ |

**Supplementary Table S3. Exclusion/inclusion criteria for quantification of autophagosomes on transmission electron microscopy analysis.**

| **INCLUSION CRITERIA** | **EXCLUSION CRITERIA** |
| --- | --- |
| Structure is ≥300nm in diameter [1] | Structure is ≤300nm in diameter |
| Structure contains cargo [1-3] | Structure is completely empty [4] |
| Structure is near, but not touching, lysosome [4] | Structure is fusing with lysosome or contains lysosomal contents |
| Double membrane is present [1, 3, 5] | No defined membrane can be seen |
| Structure is inside of cell | Structure is outside of cell |
| More than half of the structure is visible in the image | More than half of the structure is not visible in the image |
| Structure is somewhat electron dense (similar to color of cytoplasm) | Structure is extremely electron dense (lysosome) or extremely electron lucent (empty vesicle) [2] |
| Ribosomes appear inside double membraned structure (cargo) [5, 6] | Membrane is studded with ribosomes (endoplasmic reticulum) [2] |
| Electron lucent space between double membrane [1] | Cristae appear on inner membrane (damaged mitochondria) [2] |

**Supplementary References**

1. Yla-Anttila, P., et al., *Monitoring autophagy by electron microscopy in Mammalian cells.* Methods Enzymol, 2009. **452**: p. 143-64.

2. Eskelinen, E.L., *To be or not to be? Examples of incorrect identification of autophagic compartments in conventional transmission electron microscopy of mammalian cells.* Autophagy, 2008. **4**(2): p. 257-60.

3. Yoshii, S.R. and N. Mizushima, *Monitoring and Measuring Autophagy.* Int J Mol Sci, 2017. **18**(9).

4. Mizushima, N., T. Yoshimori, and B. Levine, *Methods in mammalian autophagy research.* Cell, 2010. **140**(3): p. 313-326.

5. Eskelinen, E.L., et al., *Seeing is believing: the impact of electron microscopy on autophagy research.* Autophagy, 2011. **7**(9): p. 935-56.

6. Carames, B., et al., *Autophagy activation by rapamycin reduces severity of experimental osteoarthritis.* Ann Rheum Dis, 2012. **71**(4): p. 575-81.

**Supplementary Figure Legends**

**Supplementary Figure S1. Autophagy-related proteins in the kidney.** Mice underwent either no surgical manipulations (NORM), sham surgery (SHAM), or unilateral nephrectomy (UNX). Representative immunoblots of Atg3 and Atg7 in the kidney with relative densitometry. RDU= relative densitometry units corrected for GAPDH. *P<0.05. n=4 per group.

**Supplementary Figure S2. Decreased number of lysosomes in sham surgery and UNX.** Transmission electron microscopy for lysosomes (arrow heads) with quantification is demonstrated. Lysosomes were quantified per 10µm^2^ field. Scale Bar=500 nm. *P<0.05, ***P<0.001. n=3-4 per group.

**Supplementary Figure S3. Rapamycin does not restore autophagic flux in the kidney.** Mice were treated with either vehicle or rapamycin (0.5mg/kg). Mice underwent either no surgical manipulations (NORM), sham surgery (SHAM), or unilateral nephrectomy (UNX). Each mouse was treated with either vehicle or BafA1 (BAF) and after 2 hours the contralateral kidney was harvested. Representative immunoblots of LC3-II and p62 in the kidney with relative densitometry. *P<0.05, **P<0.01. RDU= relative densitometry units corrected for GAPDH. n= 7 per group.

**Supplementary Figure S4. Metabolomics assessment of contralateral kidney. (A)** Heat map of significantly altered metabolites in normal (NORM), sham surgery (SHAM) and unilateral nephrectomy (UNX). **(B)** PCA analysis of all metabolites. **(C)** PLSDA analysis of all metabolites. Principal component analysis (PCA) and partial least squares discriminant analysis (PLSDA) were used to determine whether there were distinguishable changes in the data as a whole (Supplementary Figure S2B and C).

**Supplementary Figure S5. Specific metabolites in the kidney significantly affected by sham surgery and/or unilateral nephrectomy. (A)** Metabolites significantly decreased after both sham surgery (SHAM) and unilateral nephrectomy UNX compared to NORM. **(B)** Metabolites significantly increased only after SHAM compared to NORM. **(C)** Metabolites decreased only after SHAM compared to NORM. **(D)** Metabolites significantly increased only after UNX compared to NORM. **(E)** Metabolites significantly decreased after NEPH compared to SHAM. **(F)** Metabolite significantly decreased only after UNX compared to NORM. NHPA= 3-nitro-4-hydroxyphenylacetic acid, CDP= cytidine diphosphate, UDP= uridine diphosphate, UMP= uridine monophosphate, GMP= guanosine monophosphate, ADP= adenosine diphosphate *P<0.05, **P<0.01. n=3 per group.

**Supplementary Figure S6. Metabolites not significantly affected by sham surgery or unilateral nephrectomy.** White=NORM, light grey=SHAM, dark grey=UNX. 8**-**OHDG= 8-hydroxy-2′-deoxyguanosine, AMP= adenosine monophosphate, ATP= adenosine triphosphate, CMP= cytidine monophosphate, FAD= flavin adenine dinucleotide, IMP= inosine monophosphate, NAD(H)= nicotinamide adenine dinucleotide–hydrogen (reduced), NADP(H)= nicotinamide adenine dinucleotide phosphate-hydrogen, TMAO= trimethylamine N-oxide. n=3 per group.

**Supplementary Figure S7. Unilateral nephrectomy suppresses autophagic flux in the heart. Rapamycin inhibits mTORC1/2 in the heart but does not rescue suppressed autophagic flux in the heart after unilateral nephrectomy**. Mice underwent either no surgical manipulations (NORM), sham surgery (SHAM), or unilateral nephrectomy (UNX). Each mouse was treated with either vehicle or BafA1 (BAF) and after 2 hours the heart was harvested. (A) Representative immunoblots and relative densitometry is shown for mTORC1 (pS6) and mTORC2 (pAkt^Ser473^) substrates in the heart. *P<0.05, **P<0.01. RDU= relative densitometry units. n=4 per group. (B) Mice were treated with either vehicle or rapamycin (0.5mg/kg) before surgery. Representative immunoblots and relative densitometry is shown for mTORC1 (pS6) and mTORC2 (pAkt^Ser473^) substrates in the heart after rapamycin treatment. *P<0.05, **P<0.01. n=5 per group. (C) Immunoblot analysis of LC3-II and p62 in the heart with representative densitometry *P<0.05, **P<0.01, #P<0.0001. RDU=relative densitometry units. (n=5 per group). (D) Representative immunoblots of LC3-II and p62 in the heart after rapamycin treatment, with relative densitometry. *P<0.05, **P<0.01. n= 3 per group. RDU= relative densitometry units corrected for GAPDH.

**Supplementary Figure S8**. **Anesthesia does not suppress autophagic flux in the kidney.** (A) Mice were treated with inhaled isoflurane (ISO) for 13 minutes, injected with vehicle or bafilomycin A1, and sacrificed 2 hours later. (B) Mice were injected IP with Ketamine 80-100 mg/kg/Xylazine 7.5-16 mg/kg (K/X), injected with vehicle or BafA1 24 hours later, and sacrificed 2 hours later. Immunoblot analysis for LC3-II is shown with densitometry. n=3 per group. *P<0.05, **P<0.01, ***P<0.001.

**Supplementary Figure S1**


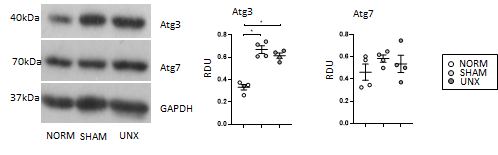


**Supplementary Figure S2**


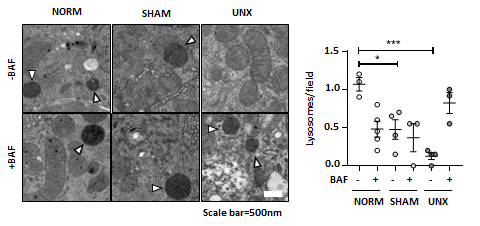


**Supplementary Figure S3**

**Supplementary Figure S4**


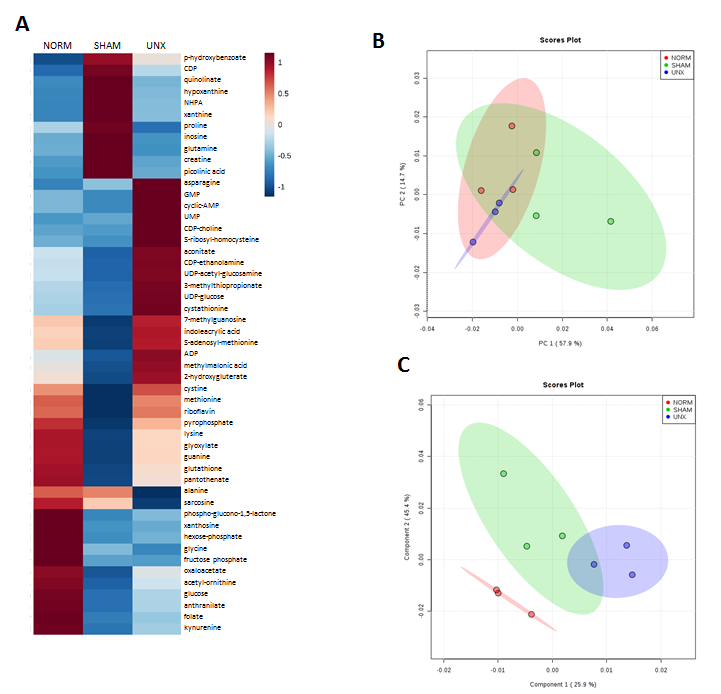


**Supplementary Figure S5**


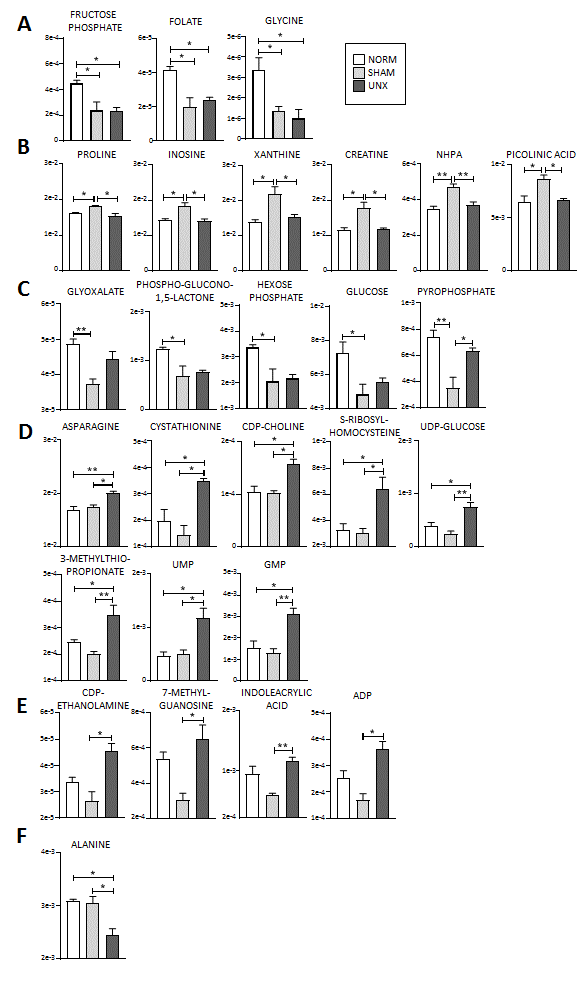


**Supplementary Figure S6**

**
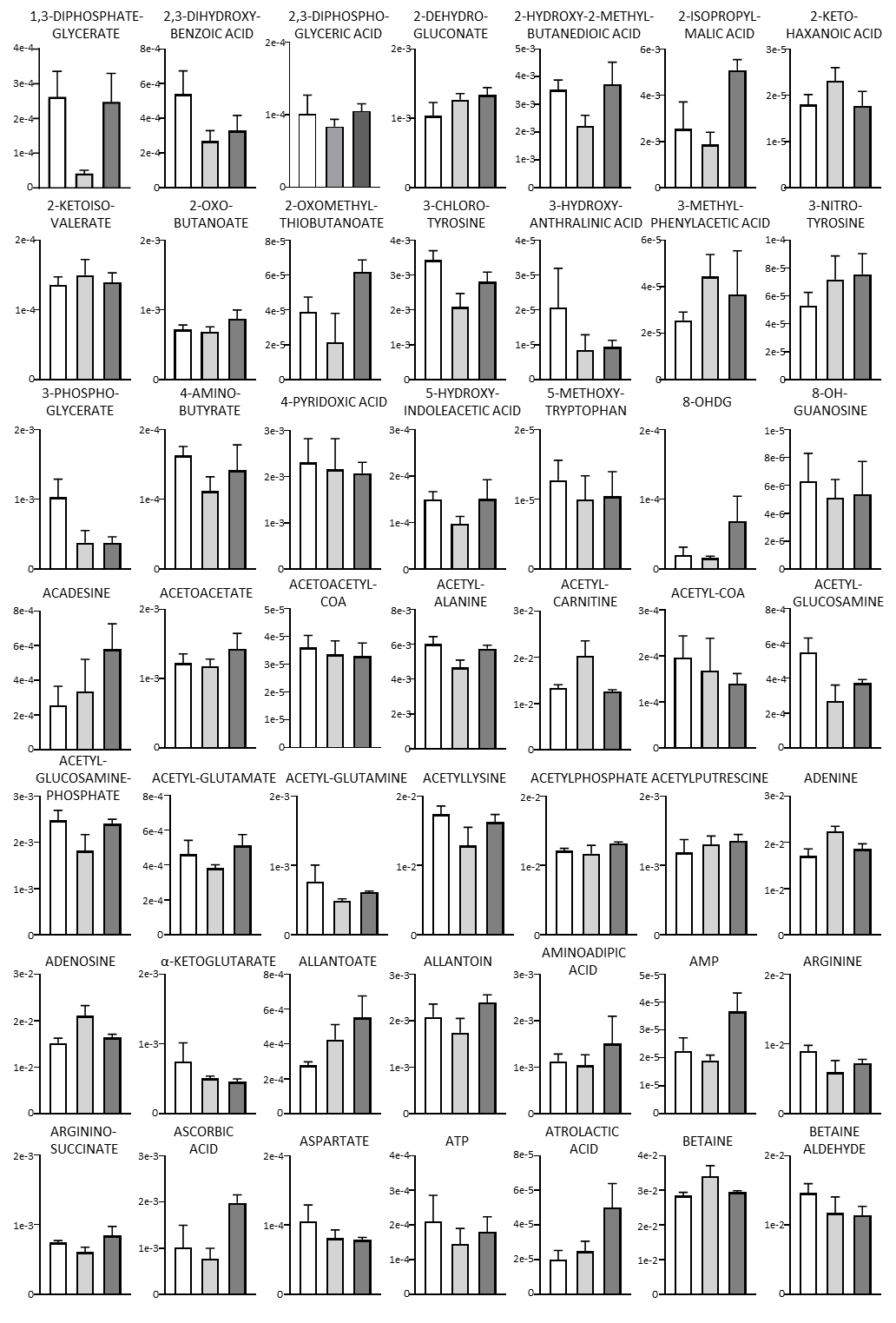
**


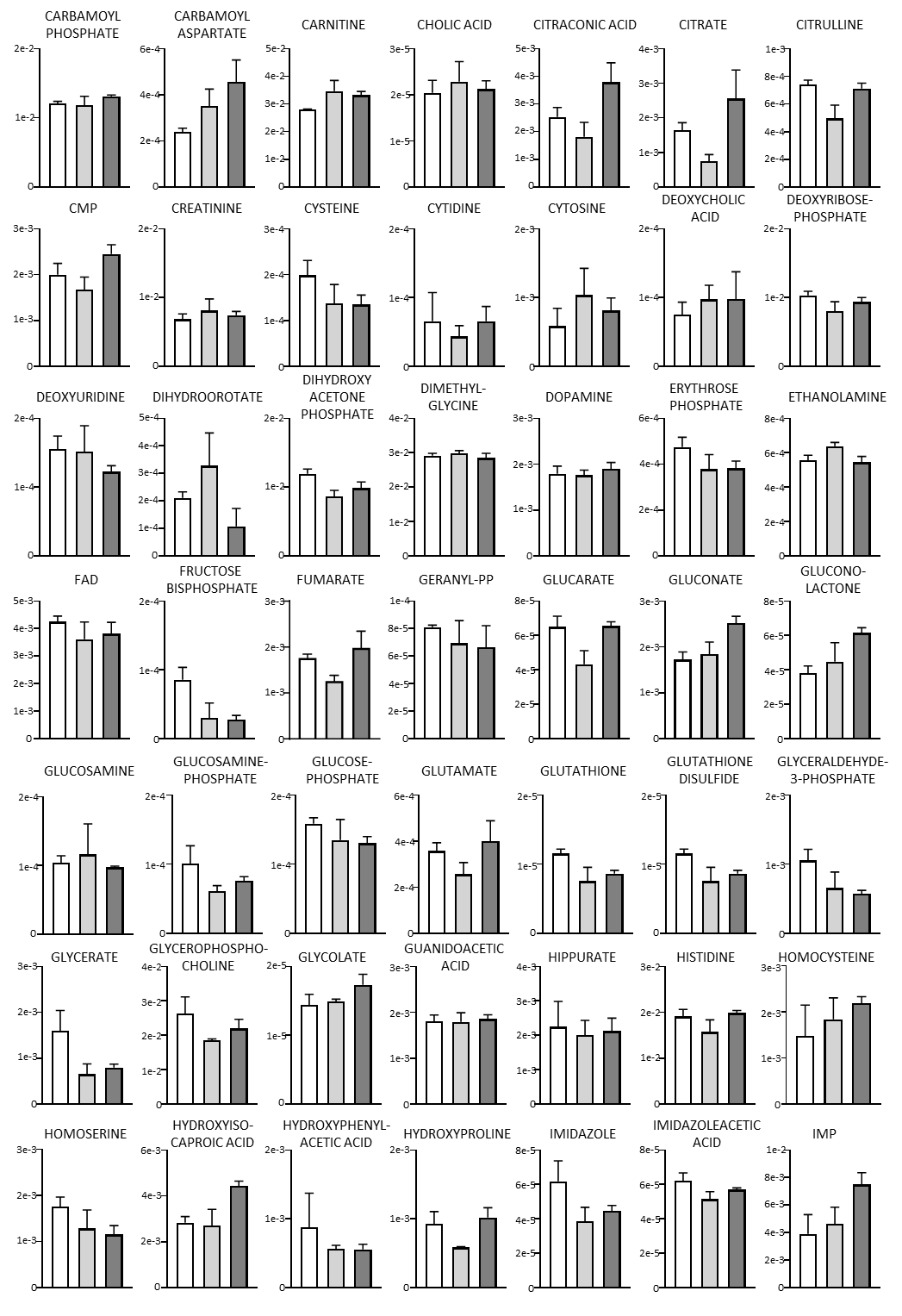


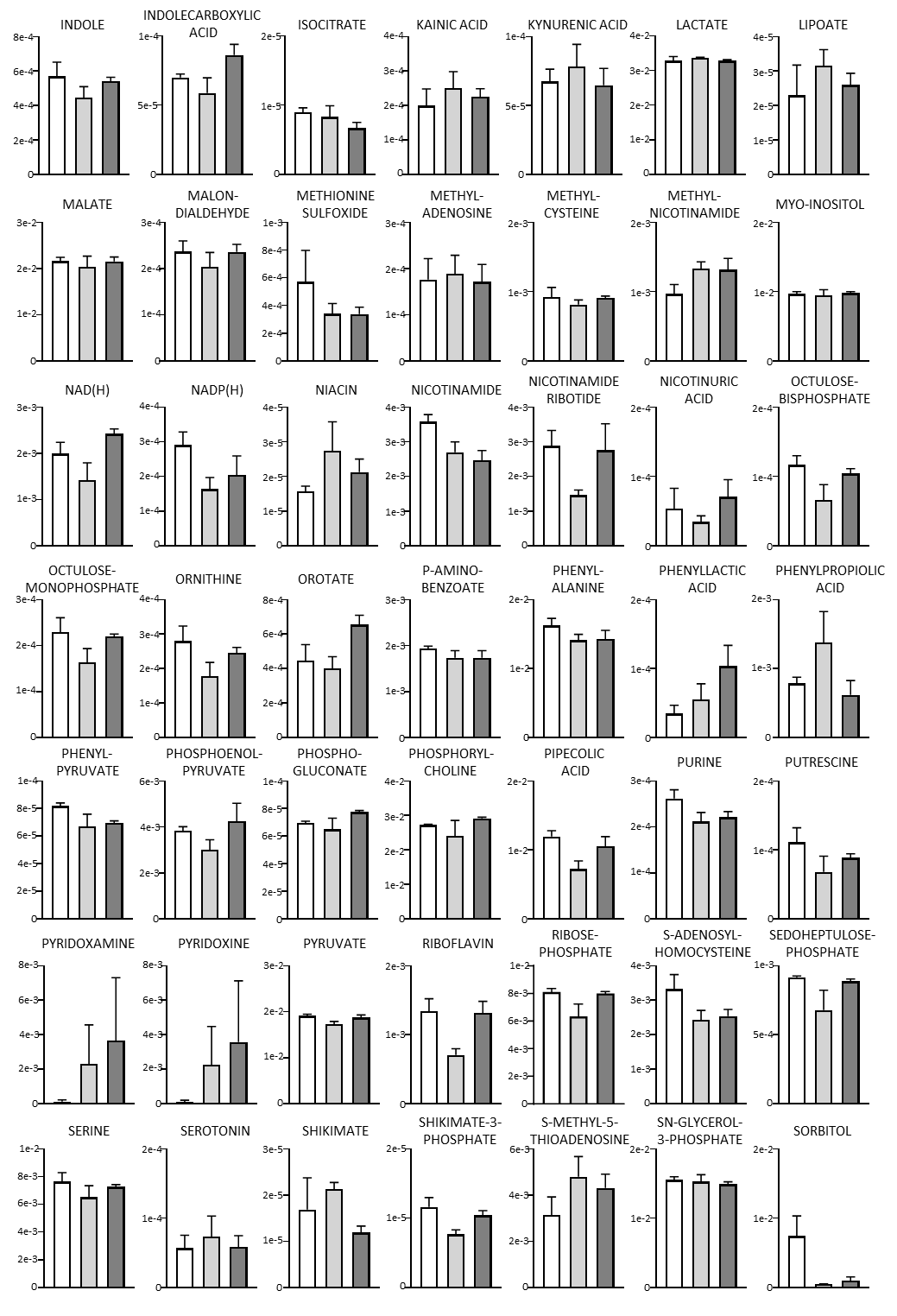


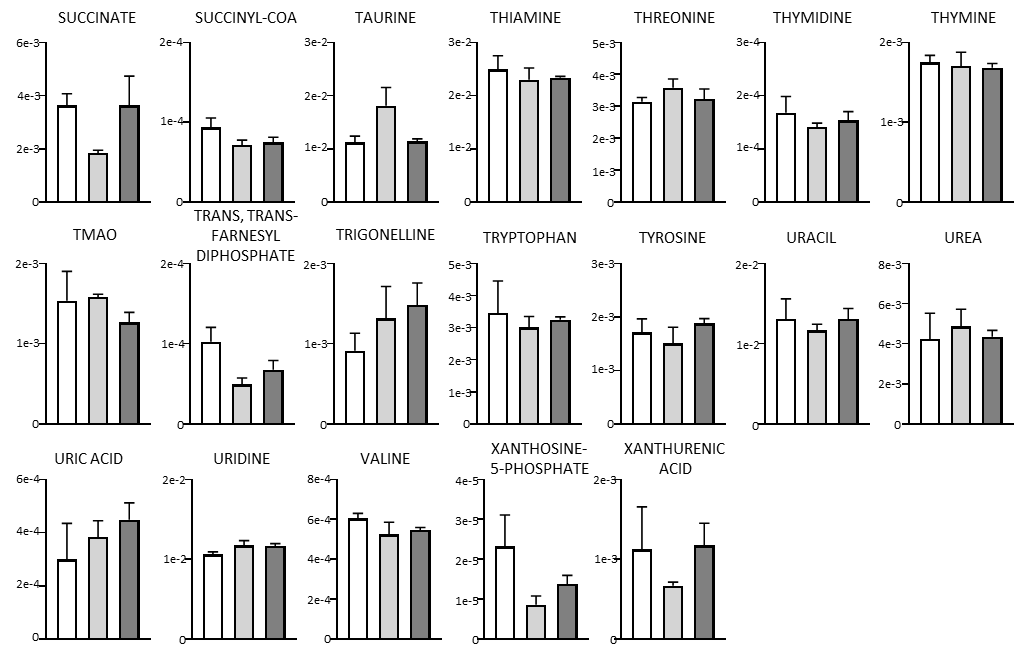


**Supplementary Figure S7**

**Supplementary Figure S8**


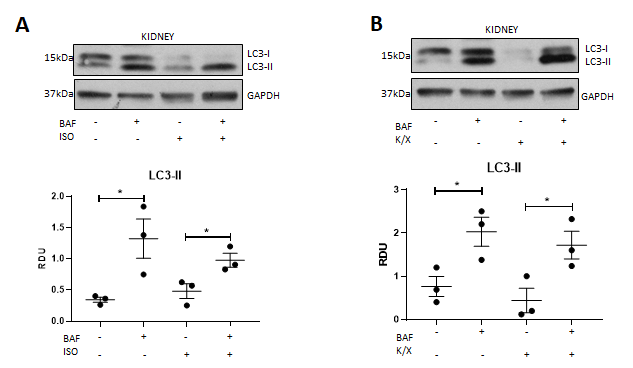

Supplement: Supplementary file 1 — Supplement [file 41419_2021_3518_MOESM1_ESM.docx]
